# Changes in mental health services and suicide mortality in Norway: an ecological study

**DOI:** 10.1186/1472-6963-11-68

**Published:** 2011-03-28

**Authors:** Håkon A Johannessen, Gudrun Dieserud, Bjørgulf Claussen, Per-Henrik Zahl

**Affiliations:** 1 Division of Mental Health, Department of Suicide Research and Prevention, Norwegian Institute of Public Health, Oslo, Norway; 2 Institute of General Practice and Community Health, Section for Social Medicine, University of Oslo, Oslo, Norway

## Abstract

**Background:**

Mental disorders are strongly associated with excess suicide risk, and successful treatment might prevent suicide. Since 1990, and particularly after 1998, there has been a substantial increase in mental health service resources in Norway. This study aimed to investigate whether these changes have had an impact on suicide mortality.

**Methods:**

We used Poisson regression analyses to assess the effect of changes in five mental health services variables on suicide mortality in five Norwegian health regions during the period 1990-2006. These variables included: number of man-labour years by all personnel, number of discharges, number of outpatient consultations, number of inpatient days, and number of hospital beds. Adjustments were made for sales of alcohol, sales of antidepressants, education, and unemployment.

**Results:**

In the period 1990-2006, we observed a total of 9480 suicides and the total suicide rate declined by 26%. None of the mental health services variables were significantly associated with female or male suicide mortality in the adjusted analyses (p > 0.05). Sales of antidepressants (adjusted Incidence Rate Ratio = 0.98; 95% CI = 0.97-1.00) and sales of alcohol (adjusted IRR = 1.41; 95% CI = 1.18-1.72) were significantly associated with female suicide mortality; education (adjusted IRR = 0.86; 95% CI = 0.79-0.94) and unemployment (adjusted IRR = 0.91; 95% CI = 0.85-0.97) were significantly associated with male suicide mortality.

**Conclusions:**

The adjusted analyses in the present study indicate that increased resources in Norwegian mental health services in the period 1990-2006 were statistically unrelated to suicide mortality.

## Background

Suicide is a complex behavioural phenomenon in which cultural, social and psychological aspects play important roles [1,2]. Thus, suicide can not be reduced to a disease [3,4]. Still, it is well documented that several mental disorders increase the likelihood of suicide [5,6]. For example, statistics from Norway reveal that approximately 15% of all who commit suicide were in specialist mental health treatment for a psychiatric disorder at the time of death [7]. Treatment of mental disorders has therefore become a key element in suicide prevention efforts, as reflected in both international guidelines from the World Health Organization [8] and several national suicide prevention programs, including the Norwegian national plan (1994) [9].

In the Norwegian democratic welfare state, public authorities are responsible for providing and financing health services [10]. The municipalities are responsible for providing primary health services and the central government is responsible for providing specialised health services. By law, all Norwegian citizens have the right to necessary and adequate health treatment and care [10].

As in most industrialised countries, mental health services in Norway have been deinstitutionalised and decentralised in recent decades [11,12]. This process entailed a shift in primary focus from inpatient to outpatient care, grounded in the assumption that decentralised and community-based mental health services would be more caring, integrative, and therapeutically successful than traditional state hospital care [13]. Likewise, in the field of clinical suicidology, the paradigm of the hospital as a necessary part of safeguarding suicidal individuals has been increasingly questioned [14]. Researchers from Finland reported a reduced suicide risk among psychiatric patients after the period of mental health deinstitutionalisation [15]. It has also been reported from Finland that municipalities with primarily outpatient and community-based mental health care were associated with lower suicide rates than municipalities with services primarily oriented towards providing inpatient treatment [16].

Not only were the mental health services in Norway deinstitutionalised, but the resources for mental health services were also considerably strengthened in quantitative terms in the period 1990-2006. Additionally, in 1998 the Norwegian Parliament adopted a national program calling for major investments in mental health services, to be implemented starting in 1999, with targets to be fully reached by the end of 2008 [17]. The expenditures in specialist mental health services had a real growth of 175% from 1998-2007 [18]. In general, investments in adult psychiatric care were allocated to increase the number of health personnel with college and university education, as well as to build more decentralised psychiatric centres and to increase the quantity and quality of inpatient and outpatient care in these centres [18]. Evaluation studies have documented that the National program has had a positive impact on patients' access to mental health services [12,18,19].

Recently, two ecological studies from the United States and Austria reported that the accessibility of mental health services [20] and the density of psychotherapists [21] were negatively associated with suicide rates. These findings need to be replicated. In Norway, no studies have addressed whether increased investments in mental health services over the past two decades have impacted the suicide rate.

The aim of this study was to determine whether increased mental health services resources had an impact on suicide mortality in five Norwegian health regions in the period 1990-2006.

## Methods

All data used in the present study are openly available and can be downloaded at Statistics Norway and Norwegian Institute for Alcohol and Drug Research except for data on sales of antidepressants, which can be provided by the Wholesales Register in Norway upon request.

In the period 1990-2006, specialist health services in Norway were organised in five health regions. Data on suicide rates in the health regions were provided by the Causes-of-Death Registry at Statistics Norway [22]. This registry has an almost complete registration of causes of death. Causes of death were classified according to the International Classification of Diseases Ninth Revision (ICD-9) during the period 1986-1995, and according to the ICD-10 from 1996 onwards.

To measure changes in mental health services, we used available health services variables provided by Statistics Norway [23]: these were the number of beds per 1000 inhabitants (beds); the number of inpatient-days per 1000 inhabitants (bed-days); the number of discharges per 1000 inhabitants (discharges); the number of outpatient consultations per 1000 inhabitants (outpatient consultations); and the number of man-labour years by all personnel in mental health services per 1000 inhabitants. Regional figures were available from 1998 onwards. For the period before 1998, annual national figures were used.

The number of inpatient-days (that is, the number of days a patient remains in hospital) is calculated by subtracting the patient's date of discharge from the date of hospital admittance [24]. The number of beds is a calculation of accessible beds in the institutions as of 31 December each year [24]. In contrast to bed-days, the number of beds is a measure of the institutions' treatment capacity [24]. The number of discharges is the number of administrative episodes; the same patient can be discharged more than once in a year [24]. Outpatient consultations "include consultations carried out in outpatient clinics or in psychiatric institutions, giving reimbursement from the state" [24]. The total number of man-labour years is estimated as: "the number of full-time jobs and part-time jobs calculated as full-time equivalents adjusted for doctor-certified sickness absence and maternity leave" [24].

To adjust for competing explanatory variables, we used regional data on education and unemployment, and national data on sales of alcohol and sales of antidepressants, all of which are reportedly associated with suicide mortality [25-28]. In addition, we analysed for linear trends in the period 1990-2006.

Sales of pure alcohol in litres per inhabitant above the age of 15 for the whole country were provided by the Norwegian Institute for Alcohol and Drug Research [29]. The number of alcohol sales for the year 1998 was missing. The figure for this year was imputed as the average of the numbers in 1997 and 1999.

Sales of antidepressants were provided by the Wholesales Register in Norway. These data represent total sales to pharmacies and institutions. In Norway, drugs are grouped according to their Anatomical Therapeutic Chemical (ATC) classification [30]. The total national sales of ATC group N06A (antidepressants) was recorded, and the sales numbers were standardised as defined daily doses (DDD)/1000 inhabitants/day for the period 1990-2006. Sales figures of antidepressants are a proxy measure; we do not know whether these drugs were consumed by patients.

Annual regional figures of the proportion of men and women per 100 with college or university education [31], and annual regional figures of the proportion of men and women per 100 unemployed [32] were provided by Statistics Norway.

A power computation showed an 80% probability of detecting a reduction in the total suicide rate of 8% or more at a 5% significance level. Data were analysed by computing crude rates and Poisson regression. Separate analyses were computed for males and females. We tested each Poisson model for over-dispersion by comparing deviance with its degrees of freedom. Deviance is a measure of the discrepancy between observed and fitted values [33]. By comparing deviance with its degrees of freedom, a goodness of fit measure is provided [33]. If the goodness of fit test is significant, then the model is inappropriate [33].

We substituted missing regional data with average national data. To examine whether this procedure had unwanted bearings on the results, we fitted national models with complete data and compared the results with the regional models. In addition, to rule out the possibility of a collinearity problem, we also computed an adjusted model in which each health services variable was analysed separately.

## Results

**Figure 1:**
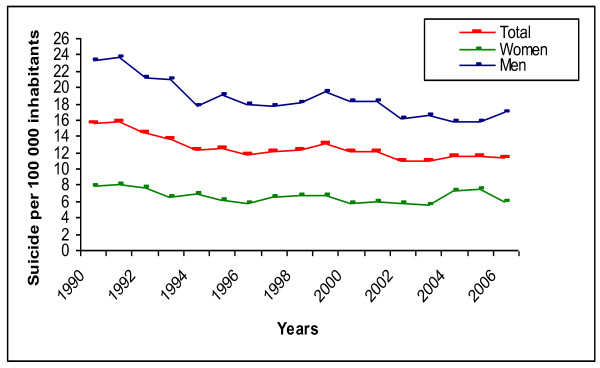
Suicide rate per 100 000 inhabitants, by sex, in the period 1990-2006

From 1990 to 2006, the total suicide rate in Norway decreased by 26% (Table 1 and Figure 1). In the same period, the number of man-labour years by all personnel, the number of discharges, and the number of outpatient consultations increased by 16%, 113%, and 222%, respectively, while the number of psychiatric hospital beds and inpatient-days declined by 42% and 43%, respectively (Table 1). Sales of antidepressants (defined daily doses (DDD)/1000 inhabitants/day) and alcohol (pure alcohol in litres per inhabitant older than 15 years of age) increased by 353% and 29%, respectively (Table 1). The proportion of unemployed individuals declined by 33% during this period, while the proportion of individuals with college or university education increased by 60% (Table 1). Regional figures are summarised in Table 2.

**Table 1:** Changes in national suicide mortality per 100 000 inhabitants

Variables	1990-1994 (RR)	1995-1999 (RR)	2000-2006 (RR)	1990-2006 (RR)
Suicide mortality	15.5-12.2 (0.79)	12.6-13.1 (1.04)	12.1-11.4 (0.94)	15.5-11.4 (0.74)
Discharges^1^	6.2-6.9 (1.11)	7.5-9.2 (1.23)	9.9-13.2 (1.33)	6.2-13.2 (2.13)
Outpatient consultations^1^	83-122 (1.47)	128-148 (1.16)	154-267 (1.73)	83-267 (3.22)
Beds^1^	2.4-2.0 (0.83)	1.9-1.7 (0.89)	1.7-1.4 (0.82)	2.4-1.4 (0.58)
Bed-days^1^	774-631 (0.82)	617-563 (0.91)	545-440 (0.81)	774-440 (0.57)
Man-labour years^1^	3.98-3.83 (0.85)	3.88-4.09 (1.05)	4.13-4.61 (1.12)	3.98-4.61 (1.16)
Antidepressants^2^	11.6-17.8 (1.53)	22.5-36.1 (1.60)	41-52.5 (1.28)	11.6-52.5 (4.53)
Alcohol^3^	4.99-4.74 (0.95)	4.79-5.45 (1.14)	5.66-6.46 (1.14)	4.99-6.46 (1.29)
Education^4^	12.2-14.2 (1.17)	14.8-16.7 (1.13)	16.9-19.5 (1.15)	12.2-19.5 (1.60)
Unemployment^5^	5.2-5.4 (1.04)	4.9-3.2 (0.65)	3.4-3.5 (1.03)	5.2-3.5 (0.67)

^1^Per 1000 inhabitants

^2^Defined daily doses (DDD)/1000 inhabitants/day

^3^Sales of pure alcohol in litres per inhabitant aged older than 15 years

^4^College and university education per 100 aged older than 16 years

^5^Unemployed per 100 (age 15-74 years)

Mortality data are accompanied by data regarding changes in explanatory variables at the national level. Rate ratios are given in parentheses.

**Table 2:** Regional figures for suicide mortality per 100 000 inhabitants in the period 1990-2006

Variables	East region (RR)	South region (RR)	West region (RR)	Mid region (RR)	North region (RR)
Suicide mortality					
Men	26.8-16.8 (0.63)	28.7-18.6 (0.68)	14.8-16.8 (1.14)	30.3-21.5 (0.71)	22.8-15.5 (0.68)
Women	10.7-6.3 (0.59)	9.3-8.1 (0.87)	4.2-5.2 (1.24)	5.2-3.9 (0.75)	7.5-5.7 (0.76)
Outpatient consultations	145-264 (1.8)	131-306 (2.3)	125-224 (1.8)	138-286 (2.1)	155-260 (1.7)
Man-labour years	4.2-5.1 (1.21)	3.4-3.8 (1.12)	4.1-4.5 (1.10)	3.9-4.2 (1.07)	4.0-4.7 (1.18)
Discharges	6.9-12 (1.74)	9.3-12.3 (1.32)	8.8-13 (1.48)	9.5-13.4 (1.41)	9-14.8 (1.64)
Beds	1.8-1.5 (0.83)	1.7-1.1 (0.65)	1.9-1.5 (0.79)	1.7-1.3 (0.76)	1.2-1.2 (1.0)
Bed-days	588-479 (0.82)	532-347 (0.65)	650-472 (0.73)	564-385 (0.68)	372-368 (0.99)
Education					
Men	16.3-21.9 (1.35)	11.6-16.3 (1.41)	12.4-17.3 (1.40)	11.2-16.5 (1.47)	9.8-14.4 (1.47)
Women	13.6-23.2 (1.70)	9.6-18.0 (1.88)	10.7-20.2 (1.89)	9.7-19.4 (2.0)	9.1-18.5 (2.0)
Unemployment					
Men	2.5-2.1 (0.84)	2.7-1.4 (0.52)	2.9-1.1 (0.38)	2.4-1.3 (0.54)	3.0-2.0 (0.67)
Women	1.4-1.3 (0.92)	1.5-1.3 (0.87)	2.0-1.2 (0.60)	1.9-1.3 (0.68)	1.8-1.4 (0.78)

Data are accompanied by regional figures for five health services variables per 1000 inhabitants in the period 1998-2006, as well as regional figures for the percentage with college and university education and regional figures of the percentage unemployed in the period 1990-2006. Rate ratios are given in parentheses.

As Figure 1 shows, the suicide rate declined the most from 1990 to 1994 (crude annual Incidence Rate Ratio = 0.94; 95% CI = 0.91-0.97) and leveled off after that (crude annual IRR = 0.99; 95% CI = 0.98-1.00). In contrast, the major changes in the health services variables were observed in the years 2000-2006 (Table 1).

A total of 9480 suicides distributed over the five health regions in Norway were observed during the period 1990-2006. As Tables 3 and 4 demonstrate, all variables that measured resources in specialist mental health services were unrelated to male and female suicide mortality when adjusted for competing explanatory factors (p > 0.05).

**Table 3:** Poisson regression models of suicide mortality among Norwegian females

Variables	Crude analysis		Adjusted analysis I^1^		Adjusted analysis II^2^	
	**IRR**^$^	**95% CI**	**IRR**	**95% CI**	**IRR**	**95% CI**

Year (1990-2006)	0.99*	0.981-0.996	1.08	0.912-1.289	1.00	0.957-1.053
Health region						
East region	1		1		1	
South region	0.98	0.883-1.084	0.74	0.301-1.831	0.98	0.883-1.085
West region	0.71*	0.638-0.800	0.56	0.300-1.058	0.71*	0.638-0.801
Mid region	0.68*	0.599-0.780	0.52	0.236-1.142	0.68*	0.599-0.779
North region	0.79*	0.682-0.905	0.57	0.234-1.400	0.78*	0.681-0.904
Outpatient consultations	1.00	0.999-1.000	1.00	0.992-1.001		
Man-labour years	1.01	0.899-1.132	1.00	0.752-1.317		
Discharges	0.97*	0.952-0.988	1.00	0.920-1.076		
Beds	1.19*	1.057-1.343	0.94	0.477-1.870		
Bed-days	1.00*	1.000-1.001	1.00	0.997-1.002		
Antidepressants	1.00*	0.993-0.999	0.97*	0.944-0.992	0.98*	0.967-0.998
Alcohol	0.95	0.887-1.011	1.76*	1.222-2.543	1.41*	1.177-1.715
Education	0.99	0.883-1.113	0.95	0.776-1.153		
Unemployment	1.00	0.990-1.013	1.11	0.914-1.339		

^$^IRR = Incidence Rate Ratio			^1^Goodness-of-fit χ^2 ^= 80.4		^2^Goodness-of-fit χ^2 ^= 84.0	
*p < 0.05			Prob > χ^2^(70) = 0.19		Prob > χ^2^(77) = 0.27	

Data are accompanied by secondary mental health services effect estimates adjusted for annual trends, health regions, sales of antidepressants, sales of alcohol, education, and unemployment.

**Table 4:** Poisson regression models of suicide mortality among Norwegian males

Variables	Crude analysis		Adjusted analysis^1^		Adjusted analysis II^2^	
	IRR^$^	95% CI	IRR	95% CI	IRR	95% CI
Year (1990-2006)	0.98*	0.973-0.982	1.07	0.988-1.159	1.02	0.996-1.045
Health region						
East region	1		1		1	
South region	1.09*	1.025-1.163	0.40*	0.200-0.799	0.46*	0.299-0.717
West region	0.79*	0.741-0.851	0.32*	0.176-0.580	0.38*	0.260-0.553
Mid region	1.25*	1.168-1.335	0.44*	0.212-0.907	0.51*	0.321-0.802
North region	1.00	0.922-1.083	0.28*	0.114-0.690	0.33*	0.184-0.581
Outpatient consultations	1.00*	0.998-1.000	1.01	0.998-1.003		
Man-labour years	0.76*	0.709-0.818	0.89	0.750-1.048		
Discharges	0.96*	0.946-0.967	1.00	0.950-1.056		
Beds	1.30*	1.215-1.399	1.15	0.785-1.700		
Bed-days	1.00*	1.000-1.001	1.00	0.999-1.003		
Antidepressants	0.99*	0.991-0.994	1.00	0.985-1.012		
Alcohol	0.85*	0.818-0.884	0.91	0.738-1.131		
Education	0.98*	0.968-0.982	0.83*	0.722-0.942	0.86*	0.785-0.940
Unemployment	1.11*	1.075-1.147	0.92*	0.859-0.985	0.91*	0.849-0.966

^$^IRR = Incidence Rate Ratio			^1^Goodness-of-fit χ^2 ^= 73.2		^2^Goodness-of-fit χ^2 ^= 79.5	
*p < 0.05			Prob > χ^2^(70) = 0.37		Prob > χ^2^(77) = 0.40	

Data are accompanied by secondary mental health services effect estimates adjusted for annual trends, health regions, sales of antidepressants, sales of alcohol, education, and unemployment.

Among women, the variables that best explained the variation in suicide mortality were health region, sales of alcohol (adjusted p = 0.01), and sales of antidepressants (adjusted p = 0.03) (Table 3). The highest suicide mortality was observed in the health region 'East,' whereas health region 'Mid' (adjusted Incidence Rate Ratio = 0.68; 95% CI = 0.60-0.78) had the lowest suicide mortality. A one-unit (one litre pure alcohol per inhabitant per year) increase in sales of alcohol predicted a 41% increase in suicide mortality (adjusted IRR = 1.41; 95% CI = 1.18-1.72), and a one-unit (one defined daily doses/1000 inhabitants/day) increase in sales of antidepressants predicted a 2% decline in suicide mortality (adjusted IRR = 0.98; 95% CI = 0.97-1.00). As can be seen by the test statistics in Table 3, the computed Poisson model had a good data fit (p = 0.27).

Among men, the variables that best explained the variation in suicide mortality were health region, education (adjusted p = 0.01), and unemployment (adjusted p = 0.01) (Table 4). The highest suicide mortality was observed in the region 'East', while health region 'North' (IRR = 0.33; 95% CI = 0.18-0.58) had the lowest suicide mortality. A one-unit (1%) increase in the proportion of men with college or university education predicted a 14% decline in suicide mortality (adjusted IRR = 0.86; 95% CI = 0.79-0.94), and a one-unit (1%) decline in the proportion of unemployed men predicted a 9% decline in suicide mortality (adjusted IRR = 0.91; 95% CI = 0.85-0.97). As can be seen by the test statistics in Table 3, the computed Poisson model had a good data fit (p = 0.40).

To control for a potential collinearity problem, we computed an adjusted model in which each of the five health services variables were included separately (not shown). None of the health services variables were statistically related to female or male suicide mortality (adjusted p > 0.05). We also computed a model with complete national data (not shown). None of the five mental health services variables were statistically associated with reduced suicide mortality for men or women (p > 0.05).

## Discussion

The aim of this study was to investigate whether increased mental health services resources had an impact on suicide mortality in the five Norwegian health regions in the period 1990-2006. Although substantial changes in mental health services resources were observed (Table 1), suicide mortality in both females and males was statistically unrelated to these changes (adjusted p > 0.05) (Tables 3 and 4).

Despite the observed decline in hospital beds, the number of discharges increased by 113% during the study period; this apparent discrepancy can be explained by the decline in the average length of hospital stays [12]. The additional increase in outpatient consultations and man-labour years by all personnel indicates that more patients were treated throughout the period. It can be argued that the shortened length of stays increases the risk of incomplete recovery, and may thereby explain the excess suicide risk generally observed in the immediate post-discharge period [34]. However, the data in the present study did not indicate increased suicide risk due to reduction in inpatient days.

Several of the adjustment variables were significantly associated with suicide mortality. Among females, increased sales of antidepressants were associated with a decline in suicide mortality (adjusted p = 0.03), while increased sales of alcohol were associated with an increase in suicide mortality (adjusted p = 0.01). Among males, increased level of college and university education were associated with a decline in suicide mortality (adjusted p = 0.01), while reduction of unemployment was associated with a decline in suicide mortality (adjusted p = 0.01). Crude differences in male and female suicide mortality were observed across the health regions. Among females, these differences do not change in the adjusted analysis; however, adjustments for educational level and the level of unemployment impact the regional differences among males. We do not know why these crude regional differences in female and male suicide mortality exist.

The present study should be interpreted with caution, because statistical associations can be masked by the fact that we may have failed to adjust for relevant confounders. Another shortcoming in the present study is that we only had one variable that directly measured the increased resources in outpatient services. Due to the process of downsizing traditional psychiatric hospitals, present mental health services policy favours active outpatient treatment. Therefore, the negative findings may be due to restricted measurements on outpatient mental health services. Further, the increased resources in child and adolescent mental health services, which were not addressed in this study, may pay off in lower suicide rates later on. Finally, it is important to be aware that associations at the individual level cannot be deduced from an ecological study design.

Our findings are in line with a recently published, cross-national, ecological study that revealed no relation between suicide rates and mental health funding, service provision, or national policies on mental health [35]. Further, in an ecological study from the United Kingdom, Lewis and co-workers [36] examined the association between standardised suicide mortality ratios and the provision of mental health services. The results demonstrated that higher quantity of provision was not negatively associated with standardised suicide mortality ratios. In addition, our findings are in line with a prospective, multi-level study from the United States, in which the researcher found no association between variation in patterns of service delivery at the system level and suicide risk [37]. However, there are ecological studies that have reported associations between various measurements of mental health services provision and suicide rates [16,20,21]. For example, Kapusta [21] and co-workers found that both sales of antidepressants and density of psychotherapists were negatively associated with suicide rates.

It is undisputable that the Norwegian mental health services have been strengthened in quantitative terms. However, little is known about the content of the treatment given and therefore the quality and effectiveness of the treatment [18]. Hence, we do not know whether more patients were successfully treated during this period. In addition, varied, interdisciplinary outpatient services that are specialised in handling suicidal patients have not been developed.

Far from all suicidal mental health patients are receiving treatments that have proven to have a preventive effect on suicide. A recent health technology assessment (review of the literature) of the effects of mental health services interventions for the prevention of suicide found that few interventions were specifically tailored to reduce suicidality [38]. Most of the studies examined in the assessment evaluated the effect of treatment related to mental illness per se. Further: "the inclusion and exclusion criteria were not always well described and in a number of studies individuals with high suicide risk were not included" [30]. This strategy, according to which suicidality is conceptualised as a symptom of mental illness and prevention of suicide requires treatment of the underlying disease, has come under increasing criticism [14,39,40]. An alternative strategy has been proposed, namely, a focus on suicidality as the primary clinical target, in which suicidal behaviour and its causes are addressed directly [14,39]. In this approach, the individual is seen as primarily suicidal with various sub-symptoms of mental illness in need of treatment [14].

Because suicide is a multi-factorial phenomenon, it is also reasonable to infer that effective suicide prevention strategies must be broader than the focus on the treatment of mental disorders. For example, public health prevention strategies that have aimed at restricting suicide means [41-43], toning down media reports [44], and restricting alcohol [45] have been demonstrably successful. Multidisciplinary approaches to suicide research and prevention are needed, which require research teams with "a balanced composition between biologically and psychologically oriented investigators" [2], and there is a "need to evaluate also other concomitant factors such as socio-economic, cultural, and religious aspects" [2].

A multidisciplinary approach does not preclude the priority of suicide prevention among psychiatric patients, who constitute a group at increased risk; however, treatment ought to target suicidality more specifically.

## Conclusions

Mental disorders are strongly associated with increased suicide risk. Therefore, successful treatment may prevent suicide, and a greater number of treated individuals may impact the suicide rate. In this study, we observed a substantial increase in mental health services resources in Norway during the period 1990-2006. However, the adjusted analyses indicate that these changes were statistically unrelated to female and male suicide mortality.

## Competing interests

The authors declare that they have no competing interests.

## Authors' contributions

HAJ made substantial contributions to the conception and design of the manuscript, drafted the manuscript, analysed and interpreted the data, and critically revised the manuscript for important intellectual content. PHZ made substantial contributions to the conception and design of the manuscript, analysed and interpreted the data, and critically revised the manuscript for important intellectual content. GD made substantial contributions to the conception and design of the manuscript, interpreted the data, and critically revised the manuscript for important intellectual content. BC made substantial contributions to the conception and design of the manuscript, interpreted the data, and critically revised the manuscript for important intellectual content. All authors read and approved the final manuscript.

## Pre-publication history

The pre-publication history for this paper can be accessed here:

http://www.biomedcentral.com/1472-6963/11/68/prepub
